# Lentinan inhibits melanoma development by regulating the AKT/Nur77/Bcl-2 signaling axis

**DOI:** 10.7150/jca.100863

**Published:** 2025-01-01

**Authors:** Xuebin Lai, Yanling Chen, Rongjie Huang, Fukai Zhu, Lanqian Huang, Nan Chen, Guipeng Li, Wenze Hou, Yutian Pan, Gulimiran Alitongbieke

**Affiliations:** 1 Engineering Technological Center of Mushroom Industry, Minnan Normal University, Zhangzhou, Fujian 363000, People's Republic of China.; 2 Department of General Surgery, Zhangzhou Affiliated Hospital of Fujian Medical University, Fujian 363000, People's Republic of China.

**Keywords:** LNT, Melanoma, Orphan receptor Nur77, Bcl-2, AKT

## Abstract

**Background:** Melanoma is a highly malignant and difficult-to-treat skin cancer. Many researchers are exploring natural products for its treatment. Lentinan (LNT), extracted from *Lentinus edodes*, exerts strong anti-tumor effects. In this study, we aimed to establish a new approach for melanoma treatment by analyzing the pharmacological properties of LNT.

**Methods:** A tumor-bearing mouse model was established to assess tumor growth. Cell survival was analyzed using the cell counting kit-8 assay. Molecular localization and expression were assessed via western blotting, histological staining, and cell staining.

**Results:** LNT significantly inhibited the growth and proliferation of melanoma cells. *In vitro*, LNT inhibited the proliferation of B16F10 cells. It also decreased the expression levels of the proliferation-related molecules, poly (ADP ribose) polymerase 1 and proliferating cell nuclear antigen, in B16F10 murine melanoma cells. Moreover, LNT decreased the expression of the orphan nuclear receptor, Nur77, but increased that of the apoptosis-related protein, Bcl-2. LNT promoted the interaction between nuclear receptor Nur77 and mitochondrial apoptosis-associated protein Bcl-2, thereby inducing apoptosis in melanoma cells. Small-interfering RNA-mediated *Nur77* knockdown revealed that LNT promoted melanoma cell apoptosis via the Nur77/Bcl-2 pathway. Furthermore, AKT played key roles in the cell apoptosis-inducing and anti-tumor effects of LNT via the Nur77/Bcl-2 pathway.

**Conclusion:** Overall, LNT inhibited tumor growth and promoted apoptosis by regulating the AKT/Nur77/Bcl-2 pathway in melanoma cells. Our findings highlight the potential of LNT for drug development and clinical treatment of melanoma.

## Introduction

Melanoma is the leading cause of death among the most aggressive and metastatic skin cancers 1, 2. Development of most melanomas is related to genetic mutations caused by long-term exposure to ultraviolet radiation 3. In 2021, there are approximately 106,110 individuals who are diagnosed with melanoma, with a mortality rate of 7% 4. Currently, surgery is the main treatment for early-stage melanoma; however, the prognosis of patients with advanced stage melanoma is dismal due to resistance to conventional treatments, including chemotherapy and radiation 2. Although various targeted treatment approaches have been developed, they do not elicit long-term clinical responses 5. Therefore, many studies are focusing on natural products to identify new therapeutics.

Lentinan (LNT), a high molecular weight β-(1,3)-glucan polysaccharide obtained from *Lentinus edodes*, is commonly known as shiitake 6. It has been extensively studied owing to its multifunctional activities 7, including anti-tumor 8, 9, immunomodulatory 10, and antiviral 11, and other biological 12, 13 activities. Recently, anti-cancer research has become more extensive owing to the increasing incidence of cancer, with LNT gaining attention in anti-tumor research 13. LNT induces apoptosis via reactive oxygen species (ROS)-mediated intrinsic and tumor necrosis factor-α-mediated external pathways 5. Additionally, it induces autophagy and apoptosis under endoplasmic reticulum stress 14. LNT also promotes the infiltration of CD4^+^ T cells into tumors and exerts indirect anti-tumor effects 15. A promising self-assembled traditional Chinese LNT nanomedicine has been established for colorectal cancer (CRC) immunotherapy 16; it also inhibits the Janus kinase/signal transducer and activator of transcription 3 signaling pathway by regulating microRNA-216a-5p, thus facilitating the apoptosis of lung cancer cells 17. However, action mechanisms of LNT against melanoma remain unknown.

Nur77, also known as TR3 or NR4A1, is a member of the nuclear receptor family 18. Nur77 performs various physiological functions in different cells and environments 18. It not only induces tumor cell apoptosis 19, 20 but also promotes tumor cell proliferation 21 and regulates cancer cell metastasis 22. Additionally, it promotes melanoma growth and metastasis 15, 16. These studies suggest the role of Nur77 in the regulation of melanoma progression. Bcl-2 is the main regulator of apoptotic pathways in human diseases, including cancer, and its protein family (e.g., Bcl-xl) can either prevent apoptosis, inducing drug resistance during cancer treatment, or promote apoptosis (e.g., Bax and Bak) 17. Interestingly, Nur77 shifts the function of Bcl-2 protein from anti-apoptotic to pro-apoptotic, facilitating the treatment of various diseases, including cancer 23-25. Therefore, we hypothesized that Nur77-Bcl-2 plays an anti-cancer role in melanoma by mediating apoptosis. LNT promotes autophagocyte death by inhibiting Nur77 expression in breast cancer model mice 26 and partly inhibits breast cancer progression via the Nur77/hypoxia-inducible factor-1α signaling axis 27. However, whether LNT affects melanoma cell apoptosis by regulating the association between Nur77 and Bcl-2 remains unknown.

In this study, we aimed to investigate whether LNT regulates the Nur77/Bcl-2 apoptotic pathway to inhibit melanoma growth. This study provides new insights into the mechanisms underlying LNT-induced tumor suppression.

## Materials and methods

### Animal treatment

Eight-week-old C57BL/6 mice (n = 28) were purchased from Shanghai Slake Experimental Animal Co., Ltd., and their dorsal hind leg skin was depilated. The mice were randomly divided into four groups: control, low-dose LNT (5 mg/kg), medium-dose LNT (10 mg/kg), and high-dose LNT (20 mg/kg) groups, with seven mice per group. B16F10 melanoma cells (approximately 2 × 10^6^ cells; 200 μL) were subcutaneously injected into the depilated area of mice. After two days of inoculation, the mice were treated with low, medium and high concentrations of LNT for 23 d, and the body weight was regularly recorded during this period. After 23 d, the mice were euthanized and subcutaneous tumor tissues were collected for observation and analysis.

### Cell culture and treatment

B16F10 cells were obtained from ATCC and cultured in the Roswell Park Memorial Institute-1640 (Xiamen, Meilun, China) culture medium at 37 ℃ and 5% CO_2_ in a humidified incubator. Approximately 10% fetal bovine serum (Pricella, Wuhan, China) and penicillin-streptomycin (100 U/mL and 100 μg/mL, respectively) were added to the Roswell Park Memorial Institute-1640 medium. Different concentrations of LNT (31.25, 62.5, 125, 250, and 500 μg/mL) were added to the B16F10 cells at 70-80% confluency, and the plates were incubated for 24 h. For MK2206 (AKT inhibitor) treatment, LNT and MK2206 (10 μM; HY10358; MCE, USA) were mixed in the medium and cultured continuously for 24 h with the cells.

### Cell transfection

B16F10 cells were grown to 60-70% confluency, and the transfection reagent system (Thermo Fisher Scientific, USA) was prepared according to the transfection quantity standard. Liposome transfection reagent Lipofectamine 3000 (Thermo Fisher) and Opti-MEM (Gibco, USA) were mixed and left at 25 ℃ for 5 min. Both solutions were transferred to the same centrifuge tube and centrifuged until both were uniformly mixed. The mixture was allowed to stand for 20 min. Subsequently, knockdown vector Nur77-shRNA, small interfering RNA targeting Nur77, and Lipofectamine 3000 were added to the cell culture dish and cultured at 5% CO_2_ and 37 ℃ for 18-48 h. After 48 h of transfection, the cells were observed under a fluorescence microscope to determine the transfection efficiency. Fluorescence reactions of the same cells under fluorescent and non-fluorescent conditions were compared.

### Western blotting

Radioimmunoprecipitation assay buffer (Beyotime, Shanghai, China) supplemented with protease and phosphatase inhibitors was used to separate the total proteins from B16F10 cells and mouse tumor tissue samples. BCA testing kit (Thermo Fisher) was used for protein quantification, according to the manufacturer's protocol. Electrophoresis was performed using sodium dodecyl sulfate-polyacrylamide gel. The proteins were transferred onto a polyvinylidene difluoride membrane (Merck Millipore, US), blocked with 5% non-fat milk for 1 h, and incubated with primary antibodies against Nur77 (12235-1-AP; Proteintech), Bcl-2 (T40056; Abmart), proliferating cell nuclear antigen (PCNA; P30108; Abmart), poly(ADP ribose) polymerase 1 (PARP1; T40050; Abmart), cleaved-PARP (AF7023, Affinity), β-actin (T0022; Affinity), AKT (10176-2-AP; Proteintech), and p-AKT (T40067; Abmart) at 4 °C overnight. The next day, the membrane was incubated with secondary antibodies (Thermo Fisher) diluted 1:5000 in TBST for 1 h. Then, ECL immunoblotting kit (Abbkine, China) was used to visualize the protein bands, according to the manufacturer's protocol.

### Immunohistochemistry

Tumor tissues of C57BL/6 mice were treated with paraffin, cut, dewaxed, and dehydrated, and the antigens were retrieved using a water bath. The sections were blocked with 0.3% H_2_O_2_ for 20 min to block the endogenous peroxidase activity. Next, the sections were incubated with 2% bovine serum albumin and primary antibodies against Nur77 (12235-1-AP; Proteintech), Bcl-2 (T40056; Abmart), and Ki67 (AF0198; Affinity) at a dilution of 1:1000 overnight at 4 °C. The next day, the sections were incubated with horseradish peroxidase-conjugated secondary antibodies (ZSGB-BIO, Peking, China), according to the manufacturer's protocol. The slices were stained with hematoxylin and observed under an optical microscope (Olympus BX51).

### Immunofluorescence (IF) assay

For cellular IF assay, the cells were cultured on glass sheets for 24 h, fixed with 4% paraformaldehyde for 15 min, and washed with phosphate-buffered saline (PBS). The adherent cells were infiltrated with 0.5% Triton X-100 and blocked for 1 h with 10% goat serum. For IF of tumor tissue, the pre-selected tissue slices were placed in the oven at 60 ℃ for 1 h for pre-dewaxing, and dewaxed in gradient alcohol. After dewaxing, the slices were placed in a citric acid antigen repair solution, and the antigen was repaired using microwaves. The tissues were circled using a histochemical pen and incubated with 3% H_2_O_2_ at room temperature for 30 min. The slices were incubated with the primary antibodies against Nur77 (12235-1-AP; Proteintech) and Bcl-2 (T40056; Abmart) at 37 °C for 60 min. After washing thrice with PBS, immunofluorescent secondary antibodies (goat anti-rabbit; 1:400) were added and the samples were incubated for 30 min at 37 °C in the dark. After washing thrice with PBS, 4',6-diamidino-2-phenylindole (DAPI) was applied to the cover sheets, and the cells were observed under an inverted fluorescence microscope (Nikon Eclipse Ti-S) at 100×.

### TdT-mediated dUTP nick-end labeling (TUNEL) assay

Tissue slices were taken from the 4 ℃ refrigerator, pre-dewaxed in a 60 ℃ oven for 1 h, dewaxed in gradient alcohol, and washed twice with PBS. Each tissue was coated with 100 μL Proteinase K solution and incubated at room temperature for 20 min. According to the TUNEL kit instructions (Yeasen, Shanghai, China), the slices were incubated with the 5× equilibration buffer for 15 min, TdT incubation buffer at 37 ℃ for 60 min, and 2 μg/mL DAPI for 5 min and washed thrice with PBS. Next, the slices were stained with DAPI, washed thrice with deionized water, and sealed with an anti-quenching agent.

### Statistical analyses

Viability data were analyzed using the ImageJ and GraphPad Prism 9 software. All statistical analyses were conducted using one-way analysis of variance for multigroup comparison and independent sample *t*-test for intergroup comparison. Statistical significance was set at P < 0.05.

## Results

### LNT inhibits the growth of subcutaneous melanoma in mice

**Figure 1:**
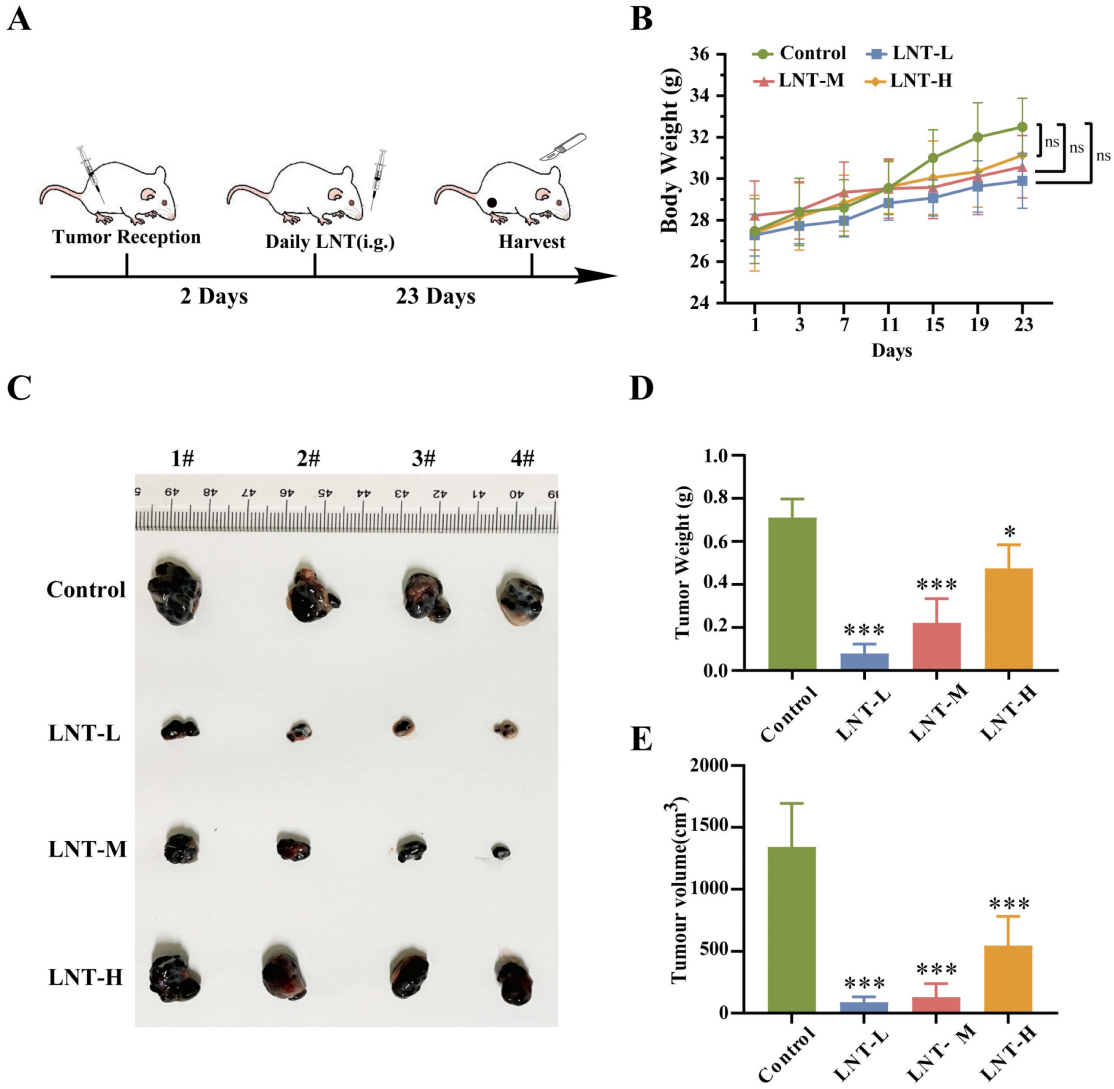
** Effect of lentinan (LNT) on melanoma tumor growth in mice. (A)** Schematic illustration of LNT treatment of melanoma model mice. The mice were randomly divided into four groups: control, LNT-L, LNT-M, LNT-H groups. **(B)** Body weight curve of mice over 23 d of treatment.** (C)** Photographs of tumors extracted from different mouse groups 23 d after modeling.** (D-E)** Tumor weight (D) and volume (E) in LNT and control groups. Data are represented as the mean ± standard deviation (SD; n = 7). Data are represented as the mean ± SD (n ≥ 3). *P < 0.05, **P < 0.01, and ***P < 0.001.

To determine whether LNT affects melanoma growth, B16F10 cells were inoculated into mice at 8 weeks of age and used for *in vivo* experiments (Fig. 1A). After modeling, tumor volume and weight were compared in the four groups. No significant differences in body weight were observed among the four groups (Fig. 1B). However, treatment with different concentrations of LNT significantly decreased the tumor size, weight, and volume compared to those in the control group, and the inhibitory effect decreased with increasing drug concentration (Fig. 1C-E). These data suggest that LNT significantly inhibits melanoma growth *in vivo*.

### LNT promotes tumor cell apoptosis in melanoma cell-bearing mice

**Figure 2:**
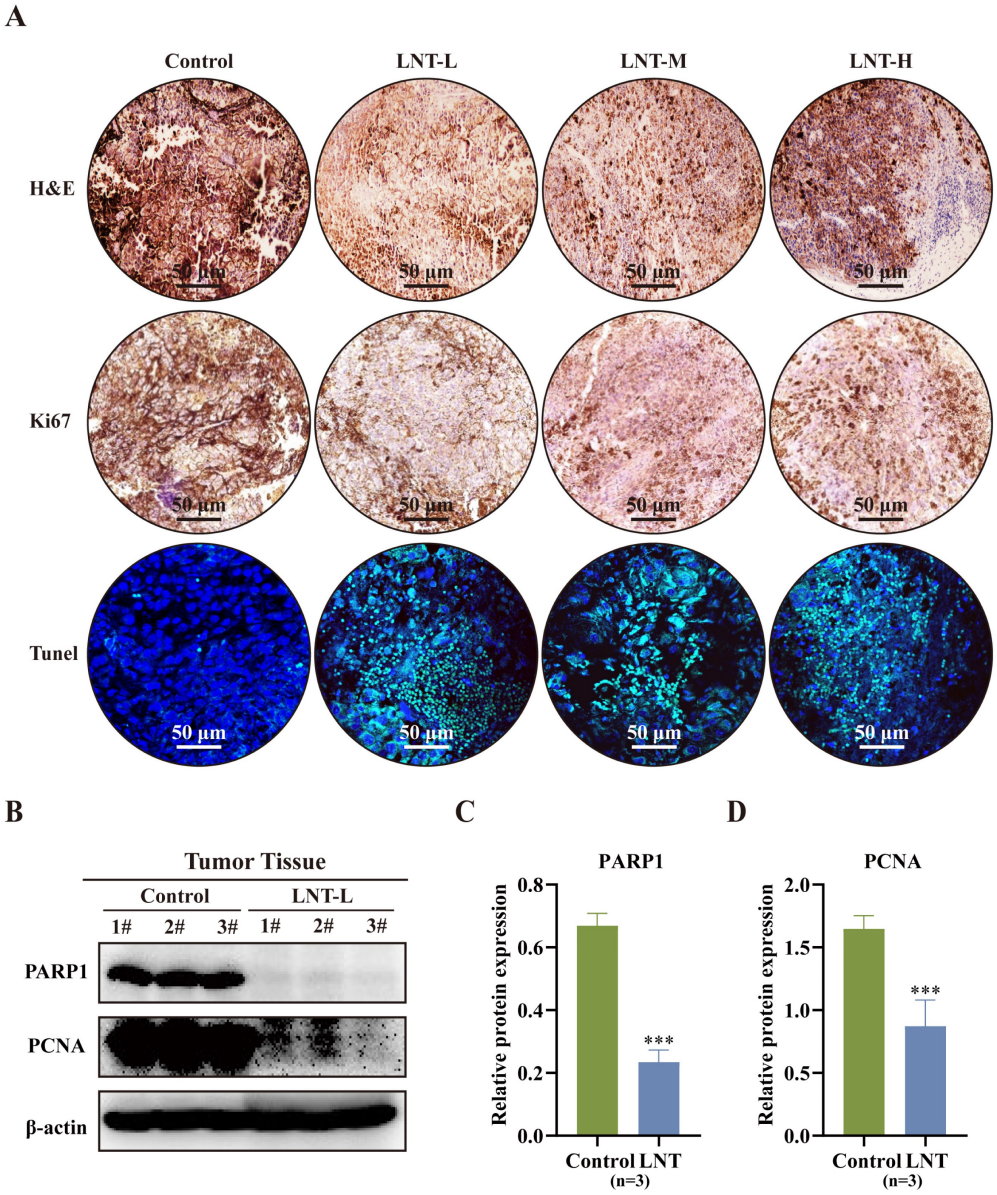
** Effect of LNT on melanoma tumor cell apoptosis. (A)** Hematoxylin and eosin (HE), Ki67, and TdT-mediated dUTP nick-end labeling (TUNEL) staining images of B16F10 tumor slices of the four groups.** (B)** Expression levels of poly (ADP ribose) polymerase 1 (PARP1) and proliferating cell nuclear antigen (PCNA) in different groups analyzed via western blotting. **(C-D)** Quantitative analysis of each protein in panel (B). Data are represented as the mean ± SD (n = 3). Data are represented as the mean ± SD (n ≥ 3). *P < 0.05, **P < 0.01, and ***P < 0.001.

Hematoxylin and eosin staining revealed that the number of inflammatory cells was lower in the LNT group than in the control group (Fig. 2A). Moreover, Ki67 and TUNEL staining revealed that different concentrations of LNT suppressed the growth of melanoma tumors and promoted cell apoptosis compared to those in the control group (Fig. 2A). Compared to those in the control group, expression levels of full-length PARP1 and proliferative marker PCNA were significantly decreased in the LNT group, indicating the anti-proliferative and pro-apoptotic effects of LNT in melanoma (Fig. 2B-D). These findings suggest that LNT inhibits the proliferation and promotes the apoptosis of melanoma cells.

### LNT induces apoptosis in B16F10 melanoma cells

**Figure 3:**
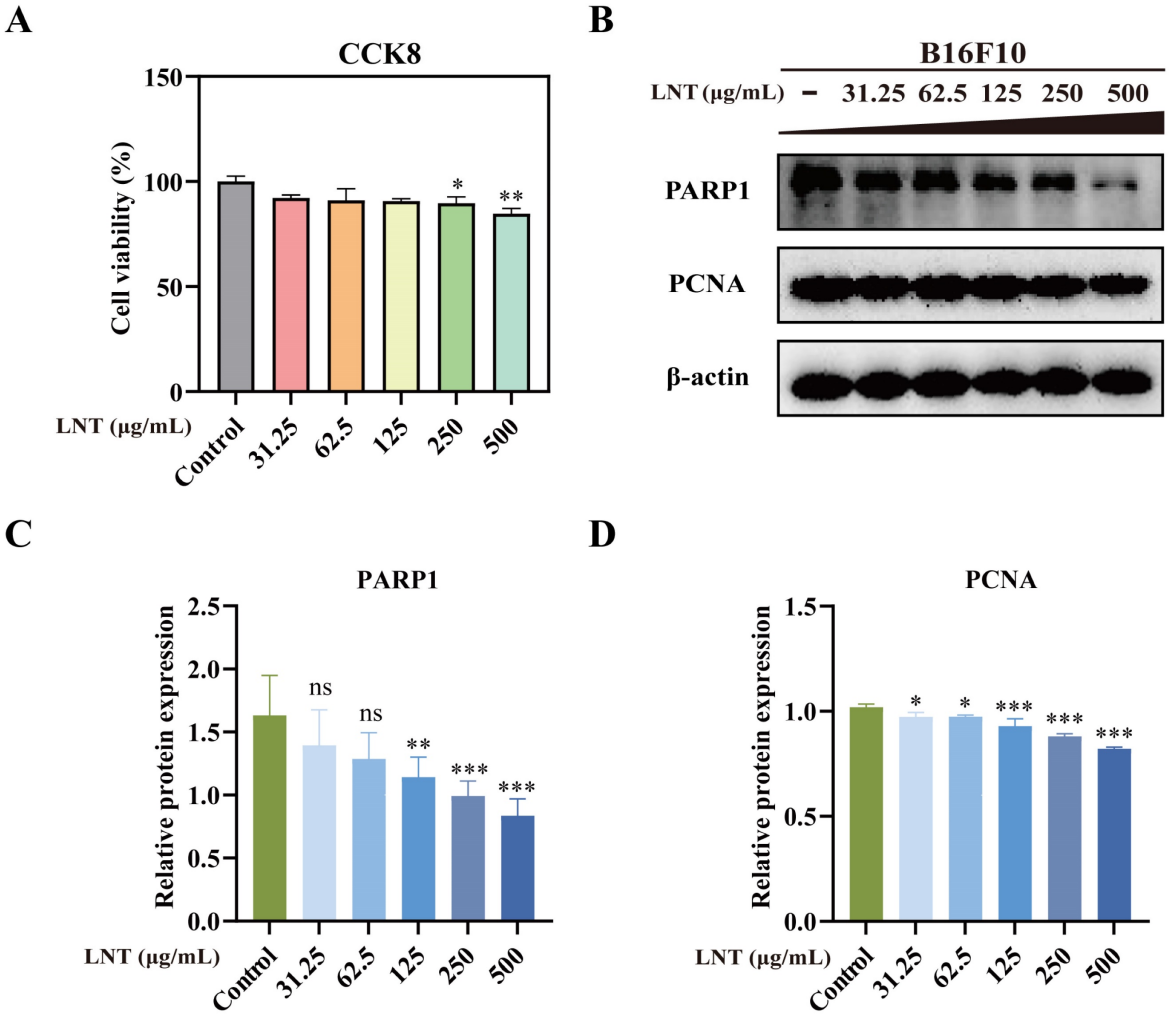
** Effect of LNT on B16F10 cell proliferation *in vitro*. (A) **Cytotoxicity of LNT at different concentrations (31.5, 62.5, 125, 250, and 500 μg/mL) assessed via cell counting kit-8 assay. **(B)** Protein levels of PARP1 and PCNA in B16F10 cells treated with various concentrations of LNT determined via western blotting. **(C-D)** Quantitative analysis of each protein in panel (B). Data are represented as the mean ± SD (n ≥ 3). *P < 0.05, **P < 0.01, and ***P < 0.001.

Cell counting kit-8 assay showed that different concentrations of LNT (31.5, 62.5, 125, 250 and 500 μg/mL), especially 250 and 500 μg/mL, significantly decreased cell viability (Fig. 3A). Western blotting also confirmed that LNT reduced the levels of full-length PARP1 and inhibited the expression of proliferation-related protein PCNA in B16F10 cells (Fig. 3B-D). These results suggest that LNT significantly inhibits B16F10 cell proliferation.

### LNT alters the expression and translocation of Nur77 and Bcl-2 in melanoma tissues

**Figure 4:**
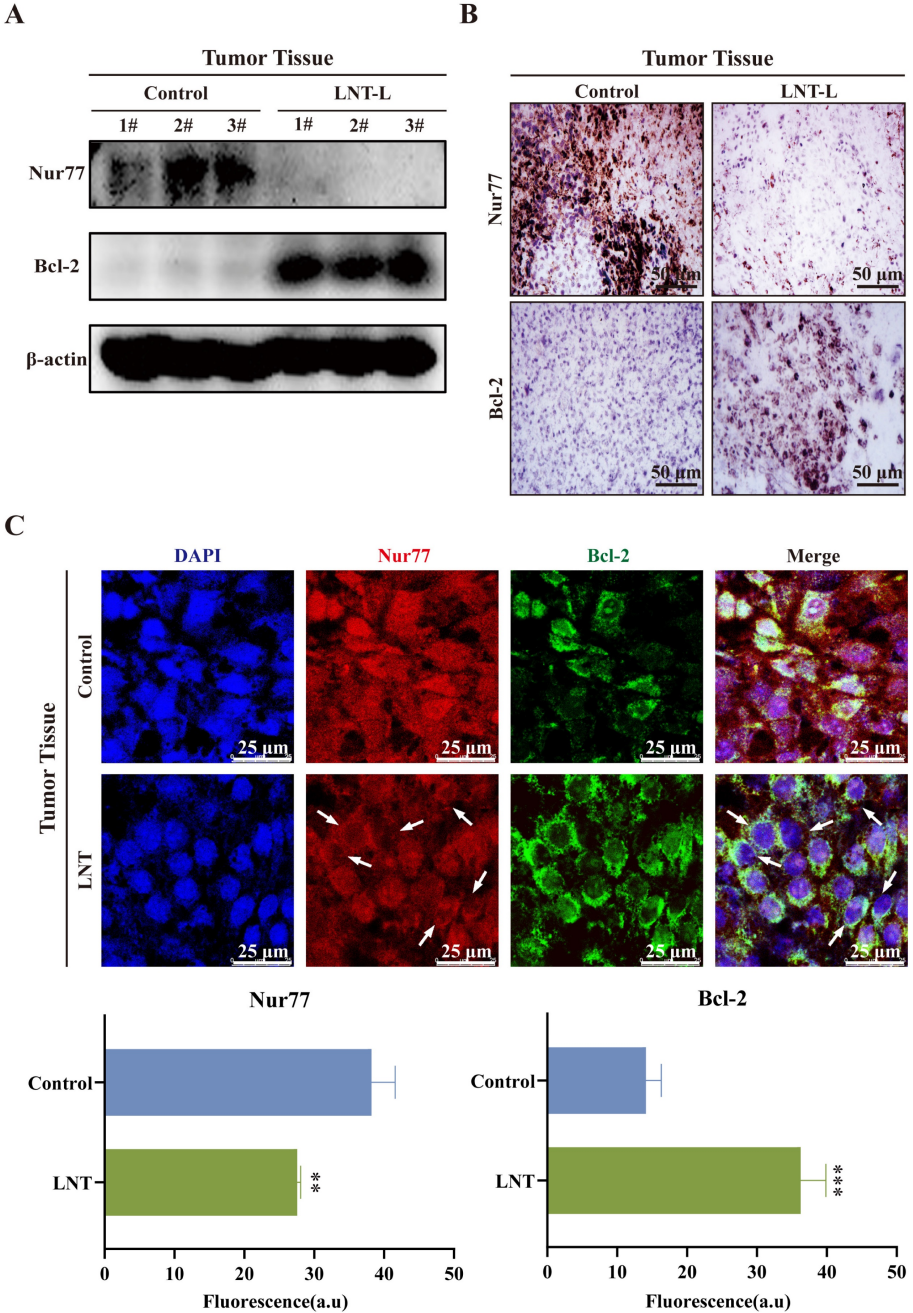
** Correlation between Nur77 and Bcl-2 in tumor tissues. (A)** Western blotting analysis of Nur77 and Bcl-2 levels in the tumor tissues of LNT-L-treated and control groups. **(B)** Positive expression of Nur77 and Bcl-2 in the tumor tissues of each group detected via immunohistochemical staining. **(C)** Fluorescence distribution and localization of Nur77 and Bcl-2 in tumor tissues determined via immunofluorescence (IF) staining. Scale bar = 10 μm. Data are represented as the mean ± SD (n ≥ 3). *P < 0.05, **P < 0.01, and ***P < 0.001.

Interaction between Nur77 and Bcl-2, which leads to the mitochondrial localization of Nur77, affects the apoptosis of tumor cells 28, 29, which is consistent with the results of this study on melanoma. Low doses of LNT decreased the protein levels of Nur77 but promoted those of Bcl-2 in tumor tissues compared to those in the control tissues (Fig. 4A). Immunohistochemical staining also indicated that the expression of Nur77 was significantly downregulated, whereas that of Bcl-2 was significantly increased after treatment with LNT in tumor tissues (Fig. 4B). Further experiments were conducted to determine whether LNT exerts anti-melanoma effects by regulating the interaction between Nur77 and Bcl-2. IF staining showed that LNT inhibited the expression of Nur77 but promoted its transport from the nucleus to the cytoplasm. LNT promoted the expression of Bcl-2 and its aggregation in the nucleus (Fig. 4C). Notably, co-localization of Bcl-2 and Nur77 in the cytoplasm was increased by LNT. Therefore, orphan nuclear receptor Nur77 is poorly expression and exhibits obvious nucleation and co-localization with Bcl-2 outside the nucleus in melanoma.

### LNT affects the expression and translocation of Nur77 and Bcl-2 in B16F10 melanoma cells

**Figure 5:**
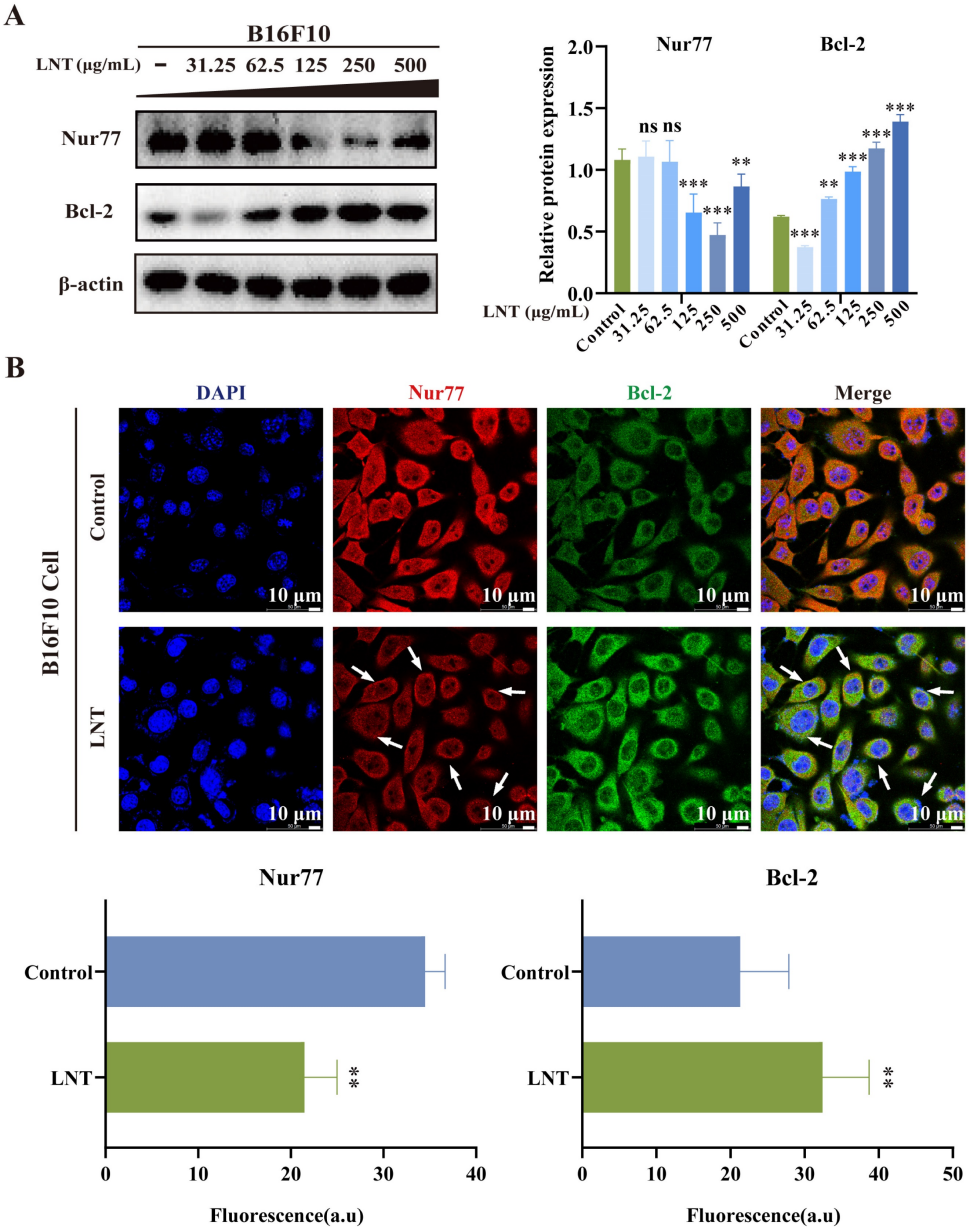
** Correlation between Nur77 and Bcl-2 in B16F10 cells. (A)** Protein expression levels and quantitation of Nur77 and Bcl-2 in B16F10 cells treated with different concentrations of LNT. **(B)** Fluorescence distribution and localization of Nur77 and Bcl-2 in B16F10 cells determined via IF staining. Scale bar = 10 μm. Data are represented as the mean ± SD (n ≥ 3). *P < 0.05, **P < 0.01, ***P < 0.001.

Overall expression of Nur77 was decreased, whereas that of Bcl-2 was increased in melanoma cells after treatment with different doses of LNT (Fig. 5A). These results indicated that LNT promoted the apoptosis of melanoma cells by affecting Nur77 and Bcl-2 expression levels. This was consistent with the results observed in melanoma tissues. Further investigation was conducted to determine the mechanisms by which LNT exerts its anti-melanoma effects via Nur77 and Bcl-2 by assessing protein co-localization. IF staining was performed on tissue slices and cells treated with LNT to observe changes in the expression and localization of Nur77 and Bcl-2. LNT inhibited the expression of Nur77 but promoted that of Bcl-2, and Nur77 showed obvious nucleation outside the cell nucleus and co-localized with Bcl-2 (Fig. 5B). These results suggest that LNT regulates the Nur77/Bcl-2 pathway by promoting Nur77 nucleation and its interaction with the mitochondrial apoptosis-related protein, Bcl-2, thereby inducing apoptosis in melanoma cells.

### LNT induces apoptosis of melanoma cells in a Nur77-dependent manner

**Figure 6:**
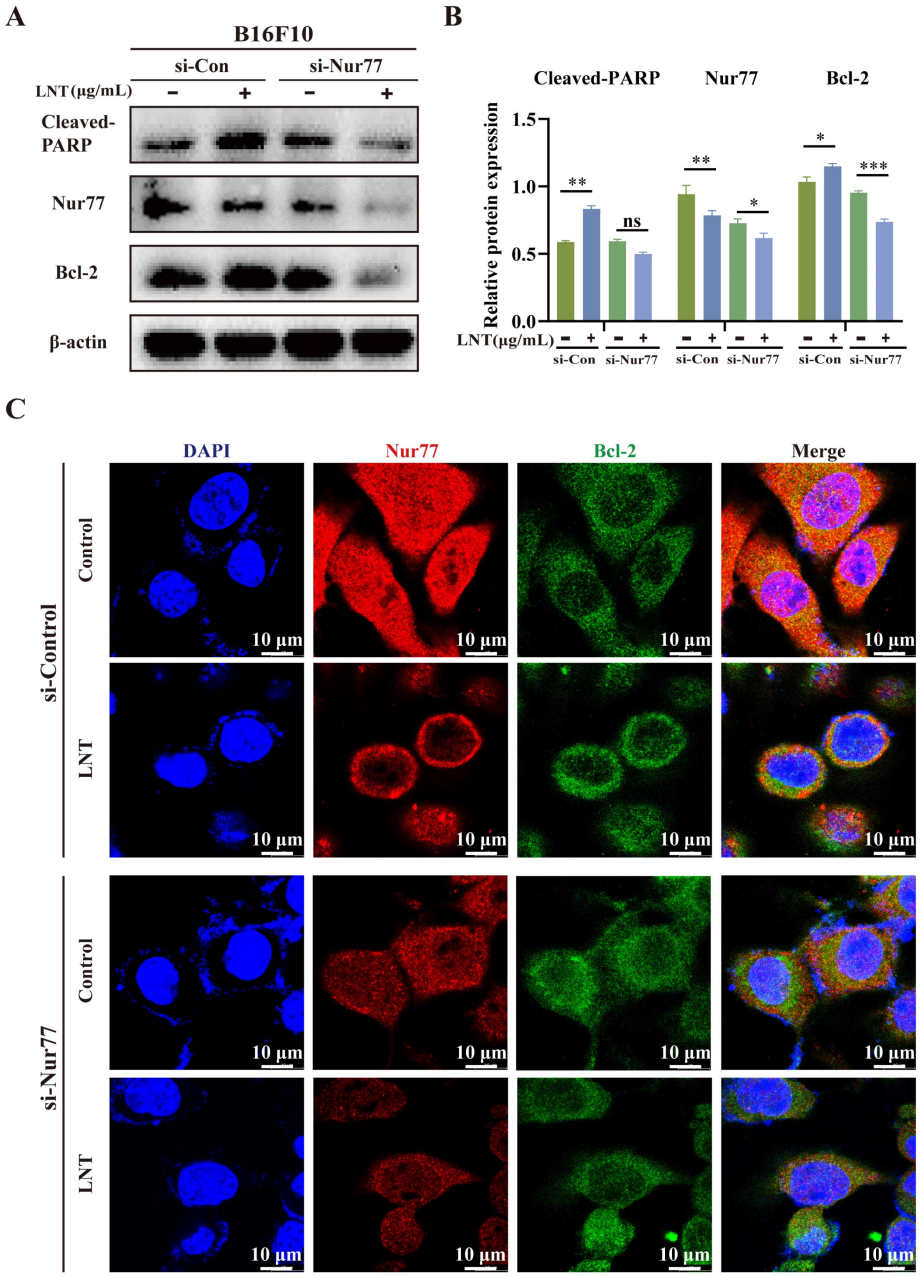
***Nur77* knockdown regulates the expression levels of Bcl-2 and cleaved-PARP.** B16F10 cells were transfected with Nur77 small interfering RNA (siRNA).** (A)** Western blotting analysis of Nur77, Bcl-2, and cleaved-PARP protein levels in B16F10 cells after transfection with si-Nur77 and empty vector with or without LNT treatment.** (B)** Protein quantitation of Nur77, Bcl-2, and cleaved-PARP in panel (A). **(C)** Fluorescence distribution and localization of Nur77 and Bcl-2 in B16F10 cells after transfection with si-Nur77 and si-control with or without LNT treatment determined via IF staining. Scale bar = 10 μm. Data are represented as the mean ± SD (n ≥ 3). *P < 0.05, **P < 0.01, and ***P < 0.001.

To confirm the role of Nur77 in the regulation of melanoma cell apoptosis by LNT, Nur77 was knocked down in B16F10 cells (Fig. 6A). LNT inhibited Nur77 expression and upregulated Bcl-2 expression and PARP cleavage. Upon endogenous Nur77 disruption, expression levels of Bcl-2 and cleaved-PARP were significantly downregulated in LNT-treated cells compared to those in control cells (Fig. 6B). IF staining also showed that LNT promoted nuclear localization of Nur77, but this phenomenon disappeared after Nur77 knockdown, which inhibited the co-localization of Bcl-2 and Nur77 in the cytoplasm (Fig. 6C). Therefore, LNT regulated Nur77, mediated Nur77 nucleation, and interacted with Bcl-2. These results indicate that LNT, associated with Nur77, inhibits melanoma development.

### LNT inhibits AKT phosphorylation in melanoma cells in a Nur77-dependent manner

**Figure 7:**
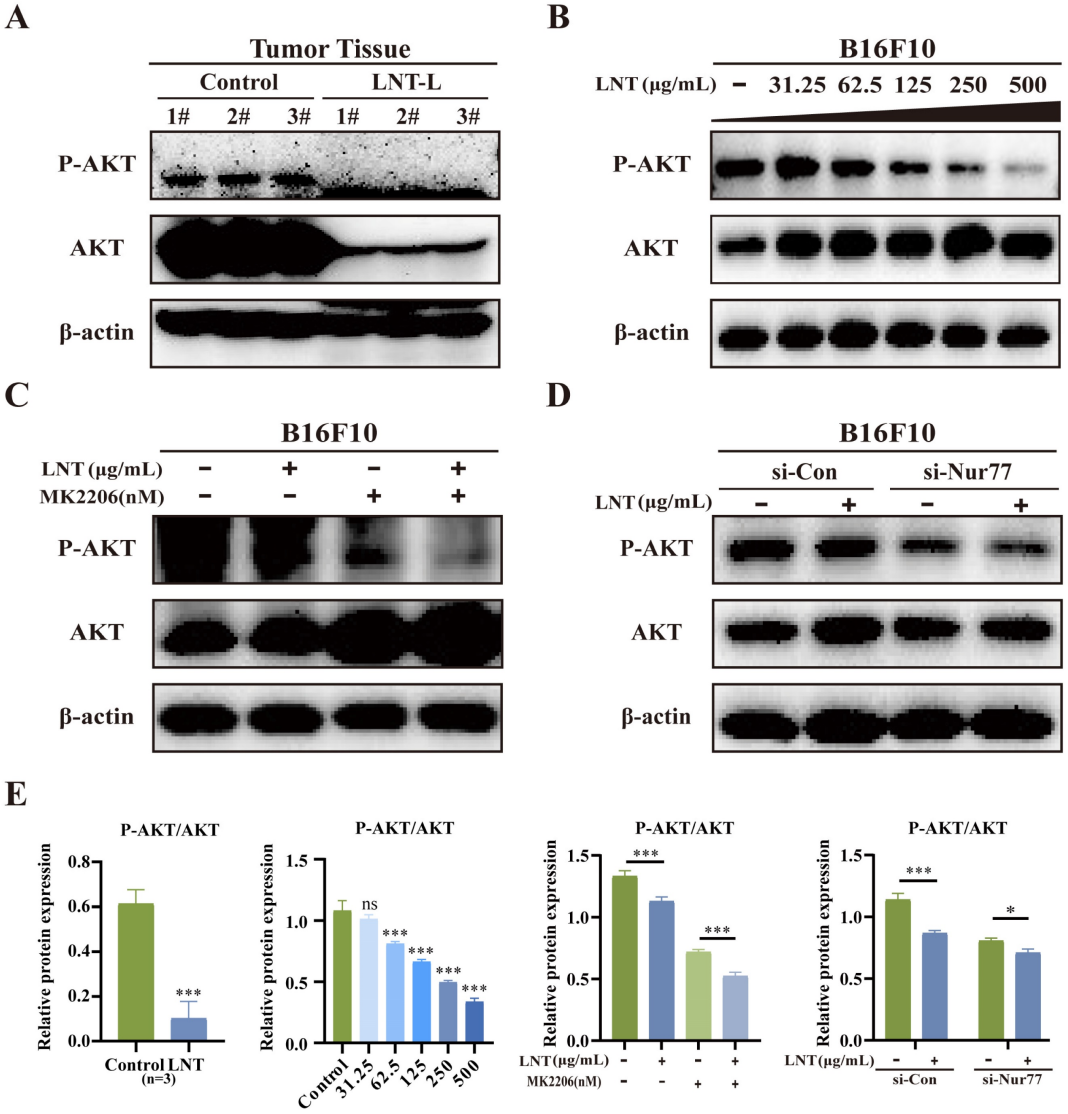
** AKT expression levels in LNT-treated tumor tissues and cells. (A)** Protein expression levels of AKT and p-AKT in LNT-L-treated and control groups determined via western blotting. **(B)** B16F10 cells in the control and LNT-treated groups were treated with MK2206. Western blotting analysis of AKT and p-AKT levels in B16F10 cells treated with different concentrations of LNT.** (C)** Protein expression levels of AKT and p-AKT in B16F10 cells treated with MK2206 and LNT. **(D)** B16F10 cells in the control and *Nur77*-knocked-down groups were treated with MK2206. Protein expression levels of AKT and p-AKT in different groups were analyzed via western blotting. **(E)** Analysis of the AKT and p-AKT ratio in panels (A-D). Data are represented as the mean ± SD (n ≥ 3). *P < 0.05, **P < 0.01, and ***P < 0.001.

LNT affects AKT phosphorylation, which further affects Bcl-2 expression. To determine whether AKT is involved in the pro-apoptotic effect of LNT on B16F10 cells, we examined the expression levels of apoptosis-related proteins. LNT inhibited the enrichment of AKT and p-AKT in tumor tissues (Fig. 7A and E) and inhibited p-AKT activity in B16F10 cells in a concentration-dependent manner (Fig. 7B and E). To further understand the roles of AKT in LNT-treated B16F10 cells, AKT inhibitor MK2206 was used in subsequent experiments. MK2206 and LNT treatment also inhibited AKT activity (Fig. 7C and E). Western blotting revealed that LNT decreased the expression of p-AKT, which was further decreased after transfection with si-Nur77 compared to that in the control group (Fig. 7D and E). These findings suggest that LNT activates AKT using Nur77 in melanoma.

### AKT mediates the interaction of Nur77 and Bcl-2 in LNT-treated melanoma cells

**Figure 8:**
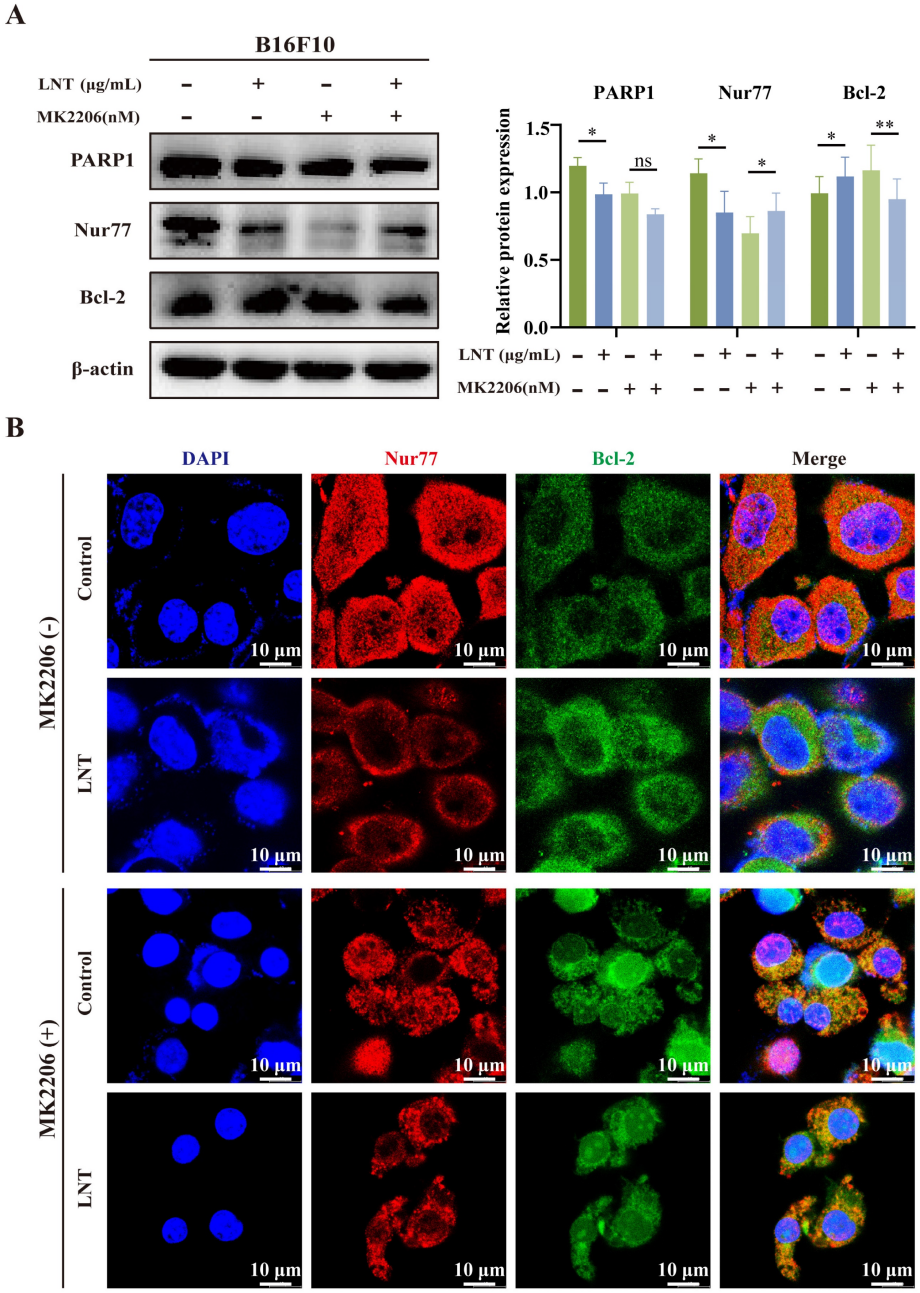
** Effect of AKT on LNT-induced apoptosis of B16F10 cells. (A)** Protein expression levels and quantitation of PARP1, Nur77, and Bcl-2 in different groups analyzed via western blotting. **(B)** Fluorescence distribution and localization of Nur77 and Bcl-2 in B16F10 cells treated with MK2206, LNT, or both. Scale bar = 10 μm. Data are represented as the mean ± SD (n ≥ 3). *P < 0.05, **P < 0.01, and ***P < 0.001.

To determine whether AKT affects the association between Nur77 and Bcl-2, melanoma cells were treated with LNT and the AKT inhibitor, MK2206. LNT inhibited the expression of full-length PARP1 but increased that of Bcl-2. MK2206 inhibited the expression of Nur77 but increased that of Bcl-2. However, MK2206 did not affect LNT-mediated downregulation of Nur77 but inhibited the expression of Bcl-2 (Fig. 8A). Additionally, MK2206 inhibited AKT activity and significantly increased the co-localization of Nur77 with Bcl-2 in the cytoplasm (mitochondria) compared to that in LNT- or LNT and MK2206-co-treated cells (Fig. 8B). These results suggest that AKT mediates the Nur77/Bcl-2 pathway in LNT-treated melanoma cells.

## Discussion

*L. edodes* is a nutritious and widely cultivated fungus, whose active extracts are widely used to treat various diseases, including cancer 30, 31. LNT inhibits the proliferation of lung cancer 32, CRC 33, and liver cancer 34. Natural compounds exhibit low toxicity, showing potential to improve the patient quality of life 35. LNT significantly inhibits melanoma growth in mice, without significant toxic effects. Additionally, LNT decreases the infiltration, proliferation, and apoptosis of melanoma cells. Therefore, LNT shows potential for melanoma treatment.

LNT induces apoptosis in Hepa1-6 cells via EGR1/PTEN/AKT signaling in hepatocellular carcinoma 30 and promotes apoptosis by targeting the Janus kinase/signal transducer and activator of transcription 3 signaling pathway in lung adenocarcinoma 36. LNT interferes with the metastasis of colon cancer and B16F10-BL6 melanoma cells to the lungs in a dose-dependent manner 26. However, the mechanism by which LNT promotes apoptosis in melanoma cells remains unclear.

Interestingly, Nur77 is highly expressed in melanoma but poorly expressed in normal tissues 37. Apoptosis is mediated by Nur77 translocation to the mitochondria 25, 38. Studies on various cancer types suggest that the mitochondrial apoptotic pathway is activated by Nur77 binding to Bcl-2 in the cytoplasm, which transforms Bcl-2 from an anti-apoptotic molecule to a pro-apoptotic molecule 23, 39, 40. Activation of the Nur77/Bcl-2 apoptotic pathway induces cancer cell apoptosis 24 and enhances the sensitivity of ovarian cancer cells to cisplatin 41. In this study, LNT decreased Nur77 expression but increased Bcl-2 expression *in vitro* and *in vivo*, and LNT-induced melanocyte apoptosis was accompanied by nuclear localization of Nur77 and its interaction with the mitochondrial apoptosis-associated protein, Bcl-2. Notably, LNT regulated Nur77, mediated Nur77 nucleation, and interacted with the mitochondrial apoptosis-associated protein, Bcl-2. However, this phenomenon was not observed on Nur77 disruption, suggesting that LNT-induced melanocyte apoptosis depends on Nur77. Therefore, nucleation of Nur77 and its binding to Bcl-2 are potential mechanisms by which LNT promotes melanoma cell apoptosis.

Key targets of SAG treatment for melanoma are enriched in the phosphatidylinositol 3-kinase/AKT pathway 42, and activating transcription factor-3 inhibits melanoma growth by downregulating the extracellular regulated protein kinase and AKT levels 43. Furthermore, Akt3 activity decreases the survival of melanoma cells, thereby inhibiting tumor development 44, 45. Association between Nur77 and Akt contributes to the invasive properties of CRC cells in hypoxic microenvironments 46. Inhibition of Nur77 by Akt induces T cell apoptosis 47. In this study, LNT inhibited AKT activity in tumor tissues and melanoma cells, suggesting that LNT inhibits melanoma growth via the AKT pathway. LNT promotes autophagy by inhibiting Nur77 expression, AKT/mammalian target of rapamycin signaling, and inflammatory signaling in breast tumor cells 26. Here, *Nur77* knockdown blocked the inhibitory effect of LNT on AKT activity as well as its pro-apoptotic effects and Nur77 inhibition. These findings indicate that AKT activity in melanoma is important for LNT-mediated promotion of cell apoptosis and Bcl-2 expression and nuclear export of Nur77 to facilitate its co-localization with Bcl-2 in the cytoplasm. Our results suggest that LNT inhibits Nur77 expression and its interaction with Bcl-2 in AKT phosphorylation-dependent manner.

## Conclusion

**Figure 9:**
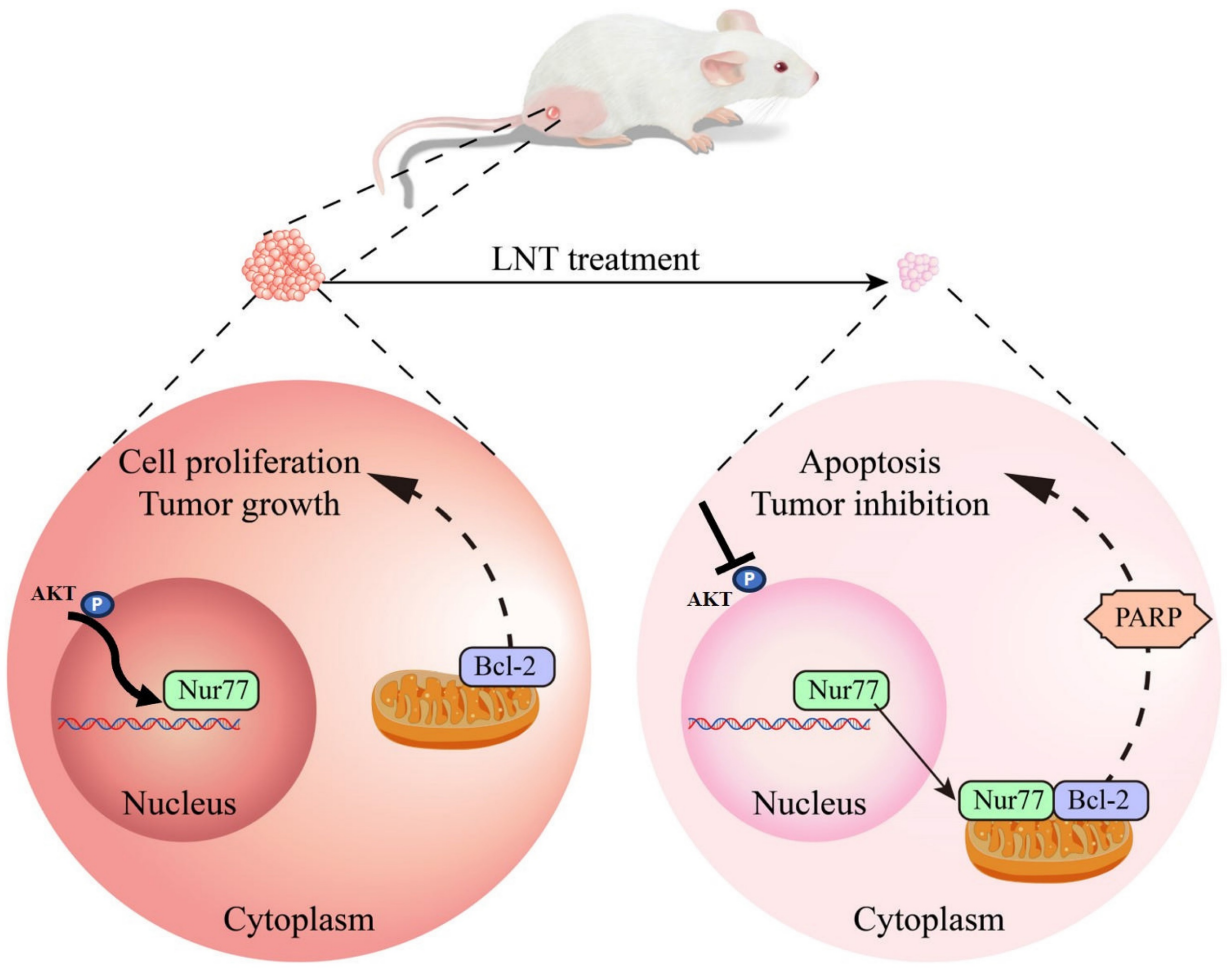
**Schematic model depicting the roles of LNT in the proliferation and apoptosis of melanoma cells.** LNT promotes apoptosis and inhibits growth of melanoma tumor cells by regulating the AKT/Nur77/Bcl-2 signaling axis.

In summary, this study showed that LNT inhibited AKT phosphorylation and affected Nur77 expression and Nur77/Bcl-2 co-localization, ultimately promoting B16F10 cell death (Fig. 9). Our results provide new insights into the mechanisms underlying melanoma inhibition by LNT. However, future studies with a larger sample size are necessary to validate our findings. Additionally, mitochondrial localization of Bcl-2 warrants further investigation.

## Abbreviations

LNT: Lentinan
ROS: reactive oxygen species
Bcl-2: B-cell lymphoma gene 2
PCNA: Proliferating Cell Nuclear Antigen
PARP: Poly ADP-ribose Polymerase
AKT: protein kinase B
